# Cylindrical Micelles by the Self-Assembly of Crystalline-*b*-Coil Polyphosphazene-*b*-P2VP Block Copolymers. Stabilization of Gold Nanoparticles

**DOI:** 10.3390/molecules24091772

**Published:** 2019-05-07

**Authors:** Maria de los Angeles Cortes, Raquel de la Campa, Maria Luisa Valenzuela, Carlos Díaz, Gabino A. Carriedo, Alejandro Presa Soto

**Affiliations:** 1 Department of Chemistry, School of Chemistry, University of Chile, 7800003 Santiago, Chile; mariadelosangeles.cortesc@gmail.com (M.d.l.A.C.); cdiaz@uchile.cl (C.D.); 2 Department of Organic and Inorganic Chemistry (IUQOEM), School of Chemistry, University of Oviedo, 33006 Oviedo, Spain; delacampafernandez@yahoo.es (R.d.l.C.); gac@uniovi.es (G.A.C.); 3 Inorganic Chemistry and Molecular Material Center, Institute of Applied Chemistry Science, School of Engineering, University Autónoma de Chile, 8900000 Santiago, Chile; maria.valenzuela@uautonoma.cl

**Keywords:** polyphosphazene, block copolymers, self-assembly, micelles, cylindrical micelles, gold nanoparticles, crystallization-driven self-assembly, P2VP

## Abstract

During the last number of years a variety of crystallization-driven self-assembly (CDSA) processes based on semicrystalline block copolymers have been developed to prepare a number of different nanomorphologies in solution (micelles). We herein present a convenient synthetic methodology combining: (i) The anionic polymerization of 2-vinylpyridine initiated by organolithium functionalized phosphane initiators; (ii) the cationic polymerization of iminophosphoranes initiated by –PR_2_Cl_2_; and (iii) a macromolecular nucleophilic substitution step, to prepare the novel block copolymers poly(bistrifluoroethoxy phosphazene)-*b*-poly(2-vinylpyridine) (PTFEP-*b*-P2VP), having semicrystalline PTFEP core forming blocks. The self-assembly of these materials in mixtures of THF (tetrahydrofuran) and 2-propanol (selective solvent to P2VP), lead to a variety of cylindrical micelles of different lengths depending on the amount of 2-propanol added. We demonstrated that the crystallization of the PTFEP at the core of the micelles is the main factor controlling the self-assembly processes. The presence of pyridinyl moieties at the corona of the micelles was exploited to stabilize gold nanoparticles (AuNPs).

## 1. Introduction

Block copolymers (BCPs) have received considerable attention due to their ability to self-assemble in both thin films (bulk) or in selective solvent to one of the blocks (solution), leading to a variety of different morphologies (e.g., spheres, rods, lamellae, etc.) [1]. In this last regard, the solution self-assembly of amphiphilic block copolymers has demonstrated to be a promising strategy to create a variety of functional well-defined core−shell nanostructures. Indeed, by controlling the solubility properties, macromolecular architectures, molecular weights, and relative volume fractions of the constituent blocks [2,3,4,5,6], micelles of different morphologies such as toroids [7], vesicles [8], disks [9,10], ovals [11], helices [12,13], and other more complex structures have been created [14,15,16,17,18,19,20,21,22]. Among these nanomorphologies, fiber-like or cylindrical micelles have been the focus of special interest due principally to their applications in drug delivery [23], as templates for inorganic nanoparticles [24,25], and as precursors to nano-patterned ceramics [26], among others [27]. Self-assembled cylindrical micelles can be prepared from coil-*b*-coil amphiphilic BCPs having significantly higher volume fraction of the core-forming block (i.e., crew-cut micelles). Importantly, these cylindrical crew-cut micelles were not very stable in solution, needing of further core or corona cross-linking treatments in order to preserve their micelle architectures [28,29]. 

Recently, Manners et al. described the formation of cylindrical micelles of different lengths (0.5–10 μm) by using block copolymers containing a semicrystalline poly-(ferrocenyldimethylsilane) (PFDMS) core-forming blocks [30]. The presence of crystallizable PFDMS at the core of the cylinder, stabilized the cylindrical micelles in solution even in the absence of cross-linking. Later, same authors exploited the crystallization of PFDMS chains to create a variety of cylindrical block co-micelles having narrow length distributions by using the so-called crystallization-driven self-assembly (CDSA) processes [31,32,33,34]. During the last years, a variety of CDSA processes have been described to create cylindrical micelles by using different semicrystalline core-forming blocks such as poly(ε-caprolactone) [35], polyacrylonitrile [36], poly(ethylene oxide) [37], polyethylene [38], enantiopure polylactide [39], and polythiophenes [40,41].

Very recently, our group synthesized a new type of crystalline-*b*-coil BCPs having a semicrystalline poly(bistrifluoroethoxy phosphazene) ([N=P(OCH_2_CF_3_)_2_]_n_, PTFEP) blocks. When these [N=P(OCH_2_CF_3_)_2_]_n_ segments were combined with polystyrene (PS) blocks (PTFEP-*b*-PS), the crystallization of [N=P(OCH_2_CF_3_)_2_]_n_ chains at the core of the micelles in solvent selective to PS blocks, led to self-assembled nano-toroids or bicontinuous nanospheres depending of the chosen experimental conditions. Moreover, we demonstrated that the degree of crystallinity of the PTFEP at the cores was responsible for the final morphology of the micelles (i.e., toroidal or bicontinuous) [42]. Although, these results evidenced the ability of semicrystalline [N=P(OCH_2_CF_3_)_2_]_n_ (PTFEP) to promote CDSA processes, cylindrical micelles were not achieved by using PTFEP-*b*-PS. Herein, we describe that while searching for BCPs having semicrystalline PTFEP chains able to produce cylindrical micelles by self-assembly, we achieved the synthesis of the new polymer PTFEP-*b*-poly(2-vinylpyridine) (PTFEP-*b*-P2VP) and studied its self-assembly in a solvent selective to the P2VP blocks. Those poly(2-vinylpyridine) chains [43], have a structure similar to that of polystyrene (PS) but the basicity and coordinative ability of the pyridinyl moieties, allow the chemical modification of the PTFEP-*b*-P2VP polymer with gold nanoparticles (AuNPs).

## 2. Results and Discussion

### 2.1. Synthesis of Telechelic Phosphane-Terminated Poly(2-vinylpyridine) (P2VP-PPh_2_, ***4***)

**Scheme 1:**
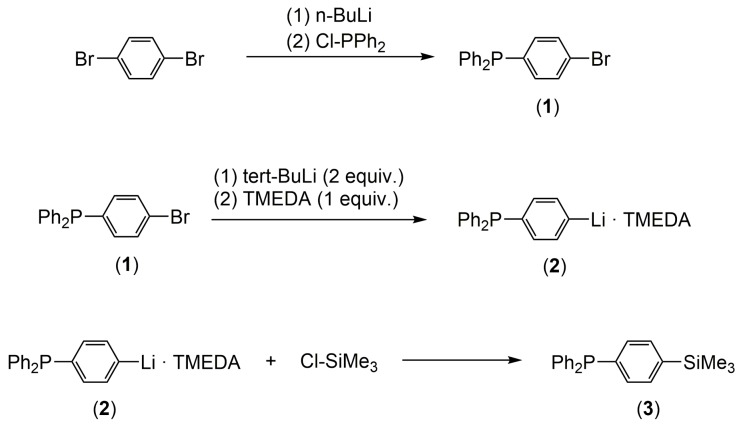
Synthesis of the diphenylphosphane-based organolithium initiator (**2**) and the (4-trimethylsilyl)diphenylphosphane (**3**).

**Scheme 2:**
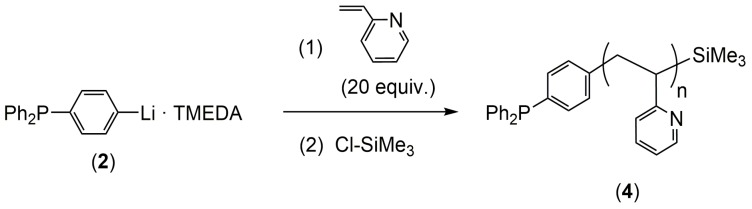
Synthesis of –PPh_2_ end-functionalized P2VP (**4**) by using organolithium substituted phosphane initiators **2**.

Currently, P2VP polymers with controlled molecular weights and narrow polydispersity indices (PDIs) can be conveniently synthesized by carbanionic or controlled radical polymerizations [43]. In this study we employed organolithium functionalized phosphane initiators to afford well-defined phosphane-terminated P2VP chains able to promote the cationic polymerization of N-trimethylsilyl phosphoranimines (Scheme 1 and Scheme 2) [44,45,46,47,48,49]. First, (4-bromophenyl)diphenylphosphane (**1**) [50] was treated with tert-BuLi and TMEDA (*N*,*N*,*N*′,*N*′-Tetramethylethane-1,2-diamine) in order to generate diphenylphosphane-based organolithium initiator species **2** (see Scheme 1 and Experimental Part). The correct formation of organolithium **2** was examined by one-pot reaction of freshly prepared solution of **2** with Cl-SiMe_3_, observing (^1^H- and ^31^P-NMR) the quantitative formation of (4-trimethylsilyl)diphenylphosphane (**3**, see Experimental Part and Supplementary Materials). It is important to note that neither the starting material **1**, nor tert-Bu-SiMe_3_, or other side-products from the reaction of un-reacted tert-BuLi and Cl-SiMe_3_ (excess), were observed. Thus, following this procedure, the lithiated phosphane initiator species **2** can be conveniently prepared in a quantitative yield in absence of any by-side reaction.

**Figure 1:**
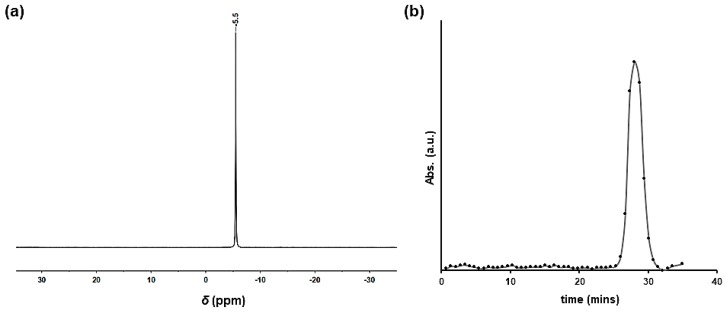
(**a**) ^31^P-NMP and (**b**) UV-GPC (Gel Permeation Chromatography with UV-vis detector) trace of polymer **4** (P2VP-PPh_2_).

Then, 2-vinylpyridine was polymerized in toluene at room temperature by using the initiator **3**. The polymerization was quenched with Cl-SiMe_3_ affording P2VP-PPh_2_ (**4**, Scheme 2). The ^31^P-NMR of **4** showed a single signal at −5.0 ppm which is in accordance with the presence of –PPh_2_ end-groups (Figure 1a). Comparison of the ^1^H-NMR integrals for the -SiMe_3_ (end-groups) protons with the polymer backbone and the aromatic pendants protons, revealed a number-average degree of polymerization of n = 24 (*M*_n_ of ca. 2900 g/mol). This was confirmed by the GPC (Gel Permeation Chromatography) analysis of **4** which gave a *M*_n_ of ca. 3000 g/mol (Figure 1b). Importantly, the observed PDI was 1.15, which is comparable to that obtained using the most common commercially available organolithium initiators (n-BuLi, sec-BuLi, etc.) [43].

### 2.2. Synthesis of Poly(Bistrifluoroethoxy Phosphazene)-b-poly(2vinylpyridine) (PTFEP-b-P2VP) Block Copolymers ***8a***–***c***

**Scheme 3:**
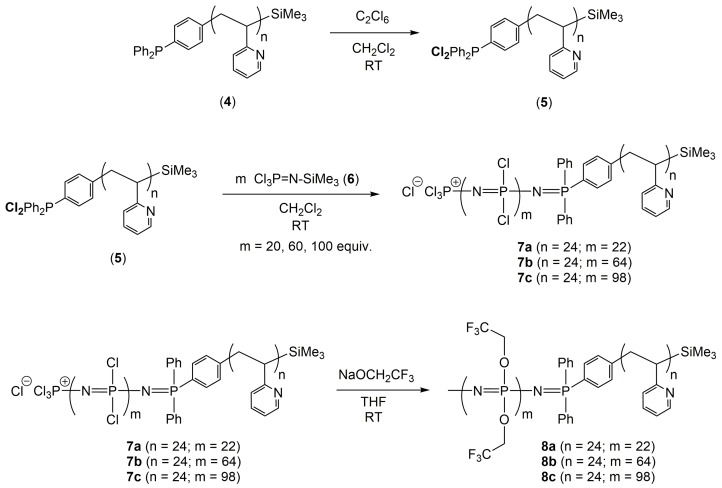
Synthesis of poly(bistrifluoroethoxy phosphazene)-b-poly(2vinylpyridine) (PTFEP-*b*-P2VP) block copolymers **8a**–**c**.

**Figure 2:**
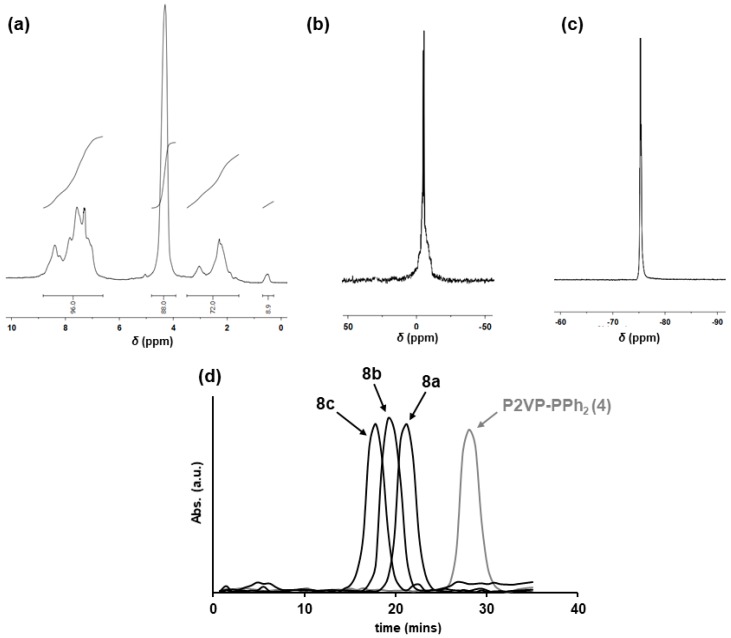
NMR spectra of BCP **8a**: (**a**) ^1^H-NMR; (**b**) ^31^P-NMR; (**c**) ^19^F-NMR. (**d**) UV-GPC trace of BCPs **8a**–**c** and polymer **4** (P2VP-PPh_2_).

It has been previously described that R_3_PCl_2_ initiates the controlled living cationic polymerization of Cl_3_P=N-SiMe_3_ (**6**) leading to polydichlorophosphazene ([N=PCl_2_]_n_) with controlled average molecular weight and narrow polydispersities (PDI < 1.3) [44,45,46,47,48,49]. Therefore, we treated P2VP-PPh_2_ (**4**) with C_2_Cl_6_ in CH_2_Cl_2_ at room temperature. The formation of P2VP-PPh_2_Cl_2_ (**5**) was demonstrated by the presence of a signal at 65 ppm in the ^31^P-NMR corresponding to the -PPh_2_Cl_2_ end-groups (see Supplementary Materials). The one-pot addition of different proportions of Cl_3_P=N-SiMe_3_ (**6**) led to block copolymers **7a**–**c** having [N=PCl_2_] segments able to be chemically modified by the macromolecular substitution of the Cl atoms with appropriate nucleophilic groups (see Scheme 3). The block ratios in BCPs **7a**–**c** were calculated by relative integration of the ^31^P-NMR signals at −17 ppm, corresponding to the (N=*P*Cl_2_) units, those of the -*P*Cl_3_ end groups at 8 ppm, and those of the Ph_2_*P*=N units at 20 ppm). The subsequent treatment of BCPs [N=PCl_2_]_n_-*b*-P2VP (**7a**–**c**) with NaOCH_2_CF_3_ led to PTFEP-*b*-P2VP (**8a**–**c**) in different block ratios and having semi crystalline [N=P(OCH_2_CF_3_)_2_] segments. The BCPs **8a**–**c** were isolated as a white solids in moderate yields (ca. 50%) with narrow polydispersities (PDI of ca. 1.2. Block ratios were calculated by relative integration of ^1^H-NMR signals of the –C*H*_2_CF_3_ protons (δ = 4.3 ppm), and those of the P2VP (Figure 2). See Table 1, Experimental Part, and Supplementary Materials for a complete description of the experimental procedures and analytical characterization details of BCPs **8a**–**c**.

The presence of [N=P(OCH_2_CF_3_)_2_]_n_ blocks was also confirmed by ^31^P-NMR and ^19^F-NMR spectra (Figure 2). The GPC analysis of **8a**–**c** gave *M*_n_ values that where higher than those obtained by relative integration on the ^1^H-NMR spectra (Table 1). This discrepancy can be accounted for by the very different hydrodynamic behavior of the PTFEP-based block copolymers and the polystyrene standards used for the GPC calibration [42,45,46]. However, although the GPC *M*_n_ values of P2VP-PPh_2_ (**4**) and BCPs **8a**–**c** are not directly comparable, it was clearly observed a consistent increase of *M*_n_ with the volume fraction (φ) of the PTFEP (see Table 1 and Figure 2). Importantly, the very narrow values of PDI (<1.2) obtained indicates that BCPs **8a**–**c** are well-defined and ideal materials for self-assembly studies [1].

### 2.3. Solution Self-Assembly of PTFEP-b-P2VP Block Copolymers ***8a***–***c***

**Figure 3:**
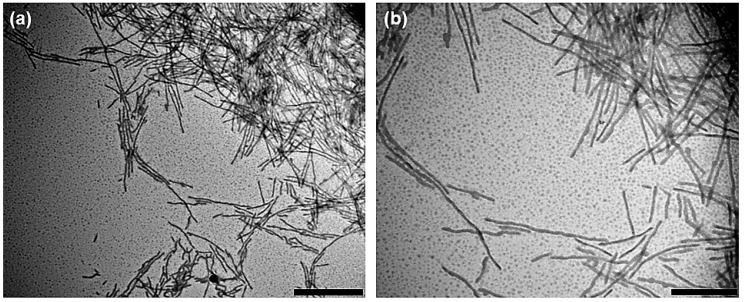
(**a**,**b**) Bright field TEM images of drop-casted solution of cylindrical micelles prepared by the self-assembly of BCP **8a** (0.33 mg/mL) in mixtures THF/2-propanol = 2/1. Scale bars correspond to 1000 nm (**a**) and 500 nm (**b**).

The self-aggregation of PTFEP-*b*-P2VP block copolymers **8a**–**c** were induced by slow addition of a solvent selective for the corona forming block over a solution in a good solvent for both blocks. This technique has been widely used as a method for preparing a variety of well-defined micelle nanomorphologies [1]. Thus, to THF solutions of BCPs **8a**–**c** (0.5 mg/mL), different proportions (hereafter indicated in % vol. relative to the starting solution) of 2-propanol (selective solvent to P2VP block) were slowly added (1 drop/5 s) and the micellization process was examined by dynamic light scattering (DLS). When the addition of 2-propanol was 10% vol. the solutions of BCPs **8b** and **8c** with higher volume fractions of PTFEP (0.8 and 0.86 respectively; see Table 1), produced a precipitate. Importantly, no variation of the values of *D*_h,App_ (apparent hydrodynamic diameter) was observed in the DLS spectrum before the precipitation. However, addition of 2-propanol over THF solution (0.5 mg/mL) of BCP **8a** having the lower volume fraction of PTFEP (0.56), did not result in precipitation of the polymeric sample. Thus, when the proportion of added 2-proponol reached the 50% vol., the *D*_h,App_ changed from 8 nm observed in pure THF (i.e., solvated free block copolymer chains or unimers) to 450 nm, denoting the formation of aggregates (micelles) in solution. These micelles were stable in solution (no change of the aggregates *D*_h,App_ values were observed within 1 week. See Supplementary Materials). The aggregates were examined by transmission electron microscopy (TEM). Thus, the micelle containing solution was directly drop-casted onto a carbon-coated copper grid, which was placed on a piece of filter paper to quickly remove the excess of solvent and minimize the drying effect on sample preparation. TEM images clearly showed cylindrical micelles having irregular or ill-defined cores (Figure 3). Cylindrical micelles with irregular cores have been previously observed in the solution self-assembly of polythiophene-*b*-poly(methyl methacrylate) BCP′s having semicrystalline polythiophene blocks [40,41]. Similarly, the formation of ill-defined cores from **8a** can be explained by considering that the crystallinity of the PTFEP block (see later the discussion about the PTFEP crystallinity), under the self-assembly conditions employed by us, affected the reversibility of micelle formation in solution, thus preventing the reorganization of the polymer chains to more regular core morphologies. These cylindrical micelles did not appear as isolated or individual objects, but were always grouped in bundles of cylinders. It was, therefore, difficult to determine the average length (*L*_n_) of the cylinders. It was clear, however, that the majority were longer than 1000 nm (note that cylinders shorter than 100 nm were also observed. See Figure 3).

**Figure 4:**
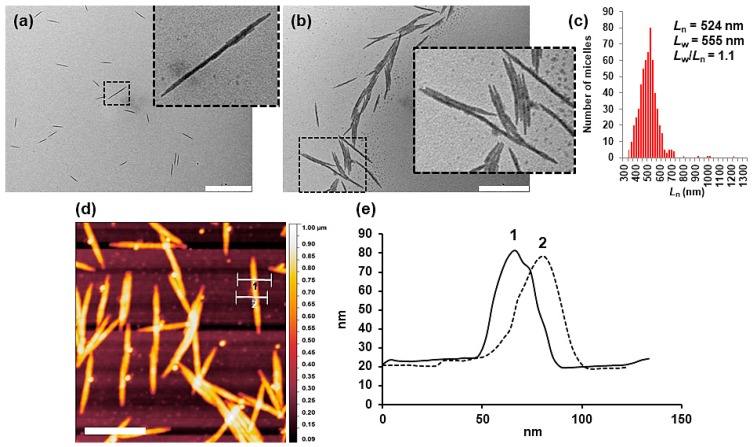
(**a**,**b**) Bright field TEM images of drop-casted solution of cylindrical micelles prepared by the self-assembly of BCP **8a** (0.25 mg/mL) in mixtures THF/2-propanol = 1/1. Inside figures correspond to higher magnification of the squared areas in (**a**) and (**b**). Inside scale bars correspond to 1000 (**a**) and 500 nm (**b**). (**c**) Histogram of micelles length (*N* = 500). AFM height image (**d**) and AFM cross-sectional height profiles (**e**) across one of the cylindrical micelles. Inside scale bar corresponds to 500 nm (**d**).

The formation of very long cylindrical micelles by addition of 50% vol. of 2-propanol suggested that, under these experimental conditions, the rate of micelle growth is higher than that of nucleation (i.e., formation of small cylindrical micelles acting as crystallization seeds). To further investigate this fact, we repeated the self-assembly of BCP **8a** by adding 2-propanol up to 100% vol. The resulting solution (THF/2-propanol = 1/1; 0.25 mg/mL) exhibited a *D*_h,App_ of 300 nm, significantly lower than the 450 nm observed with 50% vol. of 2-propanol (see Supplementary Materials). TEM images of drop-casted solutions also showed ill-defined cylindrical micelles (Figure 4a) but significantly shorter and regular in length (*L*_n_ = 524 nm; *L*_w_ = 555 nm; *L*_w_/*L*_n_ = 1.1; *N* (number of objects) = 500) than those formed with 50% vol. of 2-propanol (Figure 4c). Although the cores are also irregular, the micelles exhibited high stiffness, not observing any bending along the long axis. Similarly to the cylindrical micelles prepared from semicrystalline poly(ferrocenylsilane)-based BCPs [30,31,32,33,34], the micelles originated from **8a** tended to be aligned in the direction of the long axis, probably due to capillary forces (Figure 4b). The relative high-volume fraction of the PTFEP with respect to the corona forming P2VP in **8a**, and the stiffness of the micelles, favor the interaction of the cores and their mutual parallel orientation. Height profiles AFM (atomic force microscopy) analysis revealed an almost identical width and height (ca. 45 nm) for the as prepared micelles (Figure 4d,e), which confirmed the cylindrical geometry of the aggregates (note that the observed 2D projection on TEM can be assigned either to a cylinder or to a ribbon morphology).

**Figure 5:**
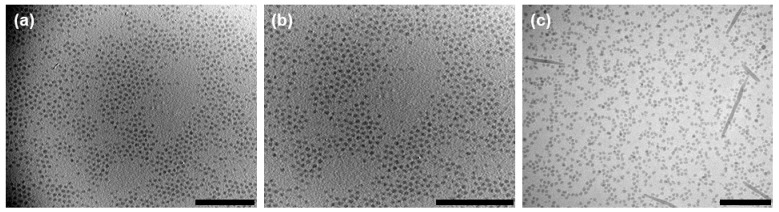
Bright field TEM images of the self-assembly of BCP **8a** at different rates of addition of 2-propanol. (**a**) 50% vol. of 2-propanol in one-step. (**b**) 100% vol. of 2-propanol in one-step. (**c**) 100% vol. of 2-propanol at the rate of 1 drop/2 s. Inside scale bars correspond to 500 nm.

The slow addition of 2-propanol (1 drop/5 s) was crucial to induce the crystallization of the core-forming PTFEP block creating cylindrical micelles. Moreover, when the solvent 2-propanol (up to 50 and 100% vol.) was added quickly (one-step) over the THF solution of BCP **8a** (0.5 g/mL), the DLS of both solutions were identical, showing an aggregation peak at *D*_h,App_ of ca. 150 nm (see Supplementary Materials). TEM images of the aggregates showed spherical micelles in both solutions (Figure 5). Interestingly, at an intermediate addition rate of 1 drop/2 s, and only when the 2-propanol added was 100% vol., mixtures of spherical and cylindrical micelles were observed by TEM (Figure 5). Although the spherical micelles were more abundant, the formation of cylindrical micelles suggested that some crystalline nucleation seeds were also formed when the addition rate was 1 drop/2 s.

Importantly, attempts to create regular cylindrical micelles by adding 100% vol. of 2-propanol over THF solution (0.5 mg/mL) of **8a** in two steps (i.e., firstly, a 50% vol. of 2-propanol was added and, after 15 min, a second portion of 50% vol. of 2-propanol was added), led to bundles of long and polydisperse cylinders micelles similar to that previously obtained when 50% vol. of 2-propanol was added. Thus, the crystallization of the PTFEP chains at the cores of the micelles, stabilizes the cylindrical morphology against further morphological evolutions.

### 2.4. Crystallization of PTFEP Block at the Core of the Micelles During the Solution Self-Assembly of PTFEP-b-P2VP Block Copolymer ***8a***

**Figure 6:**
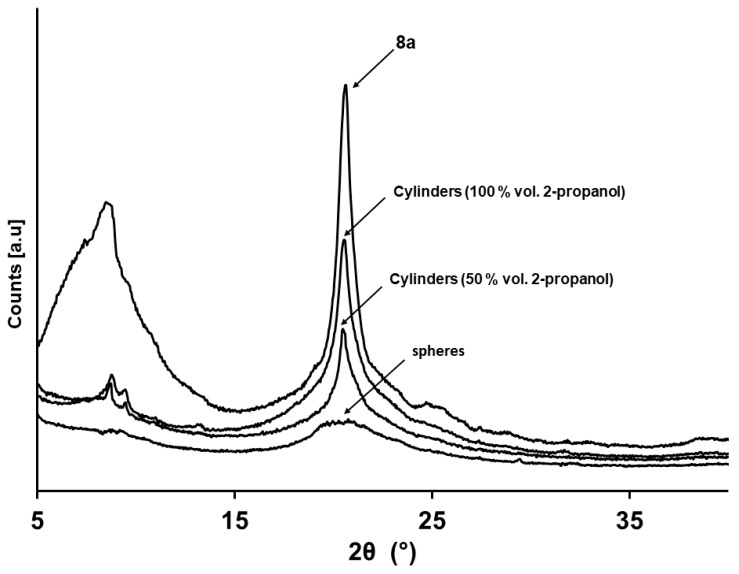
WAXS (wide-angle X-Ray scattering) diffractograms for bulk samples of BCP **8a**, cylindrical micelles from **8a** at 50 and 100% vol. of 2-propanol added, and spherical micelles resulted from the quick addition (one-step) of 100% vol. of 2-propanol.

In order to further investigate the self-assembly of the PTFEP-*b*-P2VP block copolymer **8a**, and to probe the crystallization of the PTFEP blocks at the core of the micelles, we performed wide-angle X-ray scattering (WAXS) analysis of bulk samples of block copolymer **8a** (as obtained after precipitation from solutions in THF into hexanes; see Experimental Part) and dried films containing cylindrical micelles (with 50 and 100% of 2-propanol added at an addition rate of 1 drop/5 s), or spherical micelles (50 and 100% of 2-propanol added in one-step; see Experimental Part for a complete description of the samples preparation). The WAXS experiments at room temperature of a bulk samples of BCP **8a**, showed the reflections of the crystalline domains at 2*θ* = 20.8°, and the characteristic signals corresponding to mesomorphic phases at 2*θ* = 9.028° (*d*-spacing of 9.80 Å) characteristic of the PTFEP chains (Figure 6) [42,53]. Same reflections, at 2*θ* of ca. 20 and 9°, were also observed in dried films containing cylindrical micelles prepared by adding 50 and 100% vol. of 2-propanol over THF solutions of BCP **8a** (Figure 6). All this confirmed the crystallization of the PTFEP block at the core of the cylindrical micelles. However, the degree of crystallinity of the PTFEP in the cylindrical micelles prepared from addition of 100% vol. of 2-propanol was higher than in those prepared from 50% vol. of 2-propanol. Thus, the mixture of THF/2-propanol in 1/1 ratio favored the crystallization of the PTFEP. Moreover, under these experimental conditions, the rate of nucleation and micelle growth are rather similar, which explains the very low polydispersity of the lengths of the cylindrical micelles (*L*_w_/*L*_n_ = 1.1). Additionally, the stiffness induced by the crystallization of the PTFEP chains explains both the ill-defined structure of the micelle and the absence of bending along the long axis of the cylinder. By contrast, the crystallization of the PTFEP blocks is hampered by the swelling of the chains when the proportion of the THF in the two solvents is higher, facilitating the bending of the cylinders. Moreover, the micelle growing rate, under these experimental conditions (50% vol. of 2-propanol added), is higher than the nucleation rate, which explains the longer cylindrical micelles produced. As might be expected, the quick addition of 2-propanol (one-step) precluded the crystallization of PTFEP chains leading to spherical micelles (only an amorphous halo was observed in the WAXS pattern of the film prepared from solution of spherical micelles. See Figure 6).

### 2.5. Gold Nanoparticles (AuNPs) Decorated Nanocylinders

**Figure 7:**
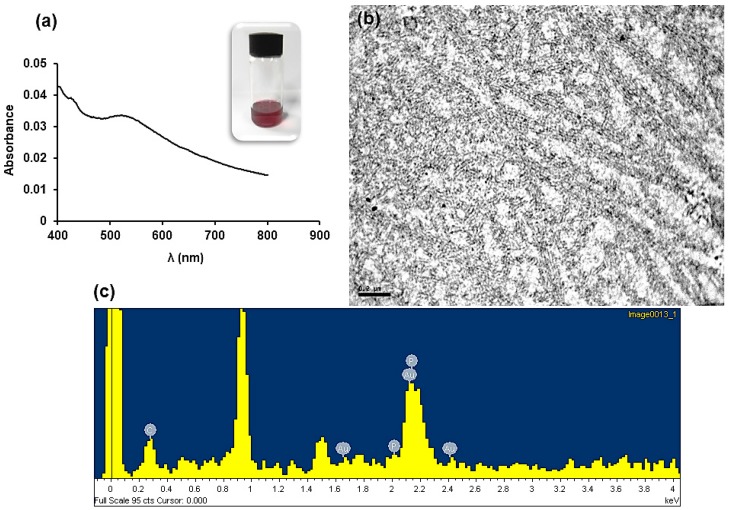
(**a**) UV/Vis absorption spectra and purple solution of cylindrical micelles of BCP **8a** containing Au NPs. (**b**) Bright field TEM image of cylindrical micelles of **8a** containing AuNPs (inset scale bar corresponds to 200 nm). (**c**) EDX analysis performed over the obtained cylindrical micelles.

The preparation of well-defined nanostructures from polyphosphazene-based block copolymers is of importance due to the applications of these polymers in biomedicine [54]. Moreover, the presence of pendant pyridinyl moieties at the corona of cylinders prepared by the slow addition of 2-propanol (100% vol.) over a THF solution (0.5 mg/mL) of block copolymer **8a**, prompted us to study the ability of these materials to be decorated with AuNPs. Thus, the solution of micelles was treated with a HAuCl_4_ (0.5 equivalent relative to the pyridine moiety). After 24 h of incubation, the solution was treated with a solution of NaBH_4_ in 2-propanol (2 equivalents relative to HAuCl_4_) [55] and a purple solution was obtained immediately (Figure 7). To rule out the presence of AuNPs not directly attached to the pyridine groups and to remove the excess of salts, the solution was purified by centrifugation for 15 min at 4000 rpm. The UV/Vis absorption spectra of the resulting clear solutions exhibited the surface plasmon resonance near to λ = 540 nm (Figure 7). Analysis of the aggregates by TEM showed tightly packed bundles of very long cylinders having stabilized AuNPs (black spots). The presence of AuNPs was confirmed by spot energy-dispersive X-ray (EDX) analysis (Figure 7; Note that also phosphorus atoms from the PTFEP chains were also detected in the EDX analysis). The presence of short P2VP coronas might cause the aggregation of the micelles in order to stabilize the formed AuNPs. Experiments performed with less equivalent of HAuCl_4_ per pyridinyl pendant group, gave similar closely packet bundles of cylindrical micelles.

## 3. Conclusions

The novel poly(bistrifluoroethoxy phosphazene)-*b*-poly(2vinylpyridine) (PTFEP-*b*-P2VP) block copolymers **8a**–**c**, having semicrystalline PTFEP core forming blocks, have been successfully synthesized by a method that proved to be of general applicability. Thus, using an organolithium functionalized phosphane initiators we polymerized 2-vinylpyridine to obtain well-defined (controlled molecular weight and narrow polydispersities) telechelic P2VP chains having –PPh_2_ end groups. The chlorination of these end-groups led to the macro-initiator P2VP-PPh_2_Cl_2_, which initiated the cationic polymerization of Cl_3_P=N-SiMe_3_. After a macromolecular substitution by an appropriate nucleophile (NaOCH_2_CF_3_), the desired PTFEP-*b*-P2VP block copolymers were achieved in moderate yield, with different block ratios, and, more importantly, with narrow polydispersities (PDI < 1.2). The self-assembly of these BCPs in a mixture of THF and 2-propanol (selective solvent to P2VP) led to cylindrical micelles of different lengths depending of the amount of the selective solvent added. Thus, whereas long cylindrical micelles with very heterogeneous lengths were achieved when 50% vol. of 2 propanol was added (i.e., 1/2 vol. over 1 vol. of the THF solution of PTFEP-*b*-P2VP), shorter and very mono-disperse cylindrical micelles were obtained when 100% vol. of 2-propanol was employed (i.e., 1 vol. over 1 vol. of the THF solution of PTFEP-*b*-P2VP). WAXS experiments demonstrated that the main factor controlling the self-assembly processes is the crystallization of PTFEP chains at the cores of the cylinder. The stabilization of AuNPs at the pyridinyl moieties located at the coronas of the micelles, produced closely packet bundles of cylindrical micelles.

## 4. Experimental Part

### 4.1. Materials

All solvents (THF, Et_2_O, CH_2_Cl_2_, toluene, and n-hexane; all from Merck, Darmstadt, Germany), were dried using an appropriate drying agent, and freshly distilled under dry atmosphere of N_2_(g) prior to being used. Toluene, for the anionic polymerization of 2-ninylpyridine, was further distilled (Na) and stored (Young’s tube) under dry Ar(g) atmosphere with molecular sieves (3 Å, 1.6 mm pellets from Aldrich, St. Louis, MI, USA) that were heated (180 °C) under vacuum (10^−2^ mbar) overnight. CH_2_Cl_2_ for cationic polymerization of Cl_3_P=N-SiMe_3_, was further distilled (CaH_2_) and stored without light (Young’s tube) under dry Ar(g) atmosphere with molecular sieves (3 Å, 1.6 mm pellets from Aldrich) that were heated (180 °C) under vacuum (10^−2^ mbar) overnight. The 2-propanol (HPLC grade, Aldrich) and the MeOH (HPLC grade, Aldrich) were used as received without further purifications. The C_2_Cl_6_ and 1,4-dibromobenzene (both from Aldrich), were purified by sublimation under reduced pressure and storage under dry atmosphere of N_2_(g). The PCl_3_, SO_2_Cl_2_, Cl-SiMe_3_, TMEDA and Ph_2_PCl (all from Aldrich), were distilled under dry atmosphere of N_2_(g) prior to being used. The 2-vynilpyridine (Aldrich) was dried with CaH_2_ and freshly distilled, and degassed, prior to be used in anionic polymerizations. The NaH (60% in mineral oil, Aldrich, stored in the glove-box), LiN(SiMe_3_)_2_ (Aldrich, stored in the glove-box), and CF_3_CH_2_OH, were used without further purification. The nBuLi (1.6 M in n-hexane) and tertBuLi (1.7 M in n-pentane), both from Aldrich, were used as received. HAuCl_4_·3H_2_O and NaBH_4_ (both from Aldrich) were used as received.

The Cl_3_P=N-SiMe_3_ (**6**) [56] and the 4-(bromophenyl)diphenylphosphine, Ph_2_P(p-C_6_H_4_-Br) (**1**), [50] were prepared according to the literature procedures.

### 4.2. Methods

All reactions were carried out under an atmosphere of either dry nitrogen or dry argon using common Schlenk techniques or a glove-box (M-Braun). The purification of the block copolymers by sequential precipitations were performed at the air by dropping a concentrated THF solution of the block copolymer onto a magnetic stirring pure water, isopropylic alcohol or MeOH, and *n*-hexanes.

NMR spectroscopy: NMR spectra were recorded at the indicated temperature on Bruker NAV-400, DPX-300 and AV-400 instruments. ^1^H- and ^13^C{1H}-NMR spectra are given relative to Si(CH_3_)_4_, ^31^P{^1^H}-NMR spectra are given relative to external 85% aqueous H_3_PO_4_. ^19^F-NMR resonances are given in relative to an external reference of CF_3_COOH.

FT-IR spectroscopy: All FT-IR spectra were recorded on a Perkin–Elmer Paragon 1000 spectrometer (PerkinElmer Inc., Waltham, MA, USA). Wavenumbers are in cm^−1^.

Elemental Analysis: The C, H, N analyses were performed with an Elemental Vario Macro instrument (Elementar, Langenselbold, Germany).

Gel permeation chromatography (GPC): GPC traces were measured with a Perkin–Elmer equipment with a model LC 250 pump, a model LC 290 UV, and a model LC 30 refractive index detector. The samples were eluted with a 0.1% by weight solution of tetra(n-buthyl) ammonium bromide in THF through Perkin–Elmer PLGel (Guard, 105, 104 and 103 Å) at 30 °C. Approximate molecular weight calibration was obtained using narrow molecular weight distribution of polystyrene standards. Samples were typically prepared by dissolving 10 mg of the polymer sample in 10 mL of micro-filtered (Millipore-Millex 0.45 µm; MilliporeSigma, Burlington, MA, USA) HPLC-grade THF. The mixture was sonicated during 5 min and magnetically stirred during 2 h. An aliquot of the solution was then filtered again (Millipore-Millex 0.45 µm; MilliporeSigma) to remove any insoluble material, and injected (20 µL) to the GPC (1 mL/min).

TEM and AFM analysis: Bright-field TEM images were obtained with a JEOL-2000-EX-II microscope (JEOL Ltd., Tokyo, Japan) operating at 160 kV and equipped with a GATAN digital camera. Contact-mode height and phase AFM images were obtained by using a Nanotec Cervantes SPM. Olympus silicon nitride AFM tips with less than 20 nm tip radius were employed.

Statistical length analysis of the nanostructures: For the statistical length analysis, nanostructures were traced manually using ImageJ software v1.52e (NIH, Bethseda, MD, USA) to determine the length of the micelles. Each TEM micrograph was analyzed completely (every single micelle in each image) in order to reduce subjectivity. From this data, *L*_n_ and *L*_w_ (average cylinder length) were calculated as shown below (number of objects (*N*) that have been traced, are indicated in the main text).

*L*_n_ = Σ *N*_i_*L*_i_ / Σ *N*_i_ (1)

*L*_w_ = Σ *N*_i_*L*_i_^2^ / Σ *N*_i_*L*_i_ (2)

Dynamic Light Scattering analysis: Dynamic light scattering measurements were performed using a Malvern Zetasizer Nano Series ZEN3600 running DTS (Dispersion Technology Software, version 6.20; Malvern Panalytical Ltd., Malvern, UK) software and operating a 4 mW He Ne laser at 633 nm. Analysis was performed at an angle of 173°and a constant temperature of 20 °C, using 1 cm glass cuvettes.

WAXS analysis: Powder XRD data were collected with Cu_Kα_ radiation (λ = 1.5418 Å) with a Bruker D8 Discover powder diffractometer fitted with a 0.4 mm fixed-divergence slit, a knife-edge collimator, and a LynxEye area detector. Data were collected in the range 2*θ* = 3–50° in *θ*/2*θ* mode.

Preparation of samples of BCP **8a** for the self-assembly studies: 0.5 mg/mL solutions of BCP **8a** in THF (typically 2 mg of **8a** in 4 mL) were prepared using micro-filtered HPLC-grade THF, and dissolved at 20 °C using an ultrasonic cleaning bath operating at 35 kHz and 160 W. Typically, a solid sample of BCP 6 was weighted in a vial using a micro balance (XPE Mettler-Toledo; Mettler Toledo, Columbus, OH, USA). Then, filtered THF was added at room temperature and the mixture was sonicated during 5 min (20 °C). The solution was analyzed by DLS to probe the absence of aggregates. At this point, and under very slow magnetically stirring conditions, the desired amount of 2-propanol was added (typically 2 mL to 50% vol., and 4 mL to 100% vol.). The addition rate was fixed to 1 drop/5 s (typically 3–4 min for 50% vol. and 6–8 min for 100% vol.). When the desired amounts of 2-propanol were quickly added in one-step, the addition time was 1–2 s. Solution was allowed to stand at 20 °C and then was studied by DLS. DLS analysis on sample solution of micelles was again analyzed by DLS after 24 h. At this point, an aliquot of the solution was examined by TEM and AFM. The samples for TEM were prepared by drop-casting one drop (ca. 10 µL) of the block copolymers solution onto a carbon-coated copper grid which was placed on a piece of filter paper to remove excess solvent. No staining of the samples was necessary. The samples for AFM were prepared by drop-casting one drop (ca. 10 µL) of the block copolymers solution (THF) onto a mica disc (10 mm, V1 Pk10, from Agar Scientific Ltd., Essex, UK).

Preparation of samples of micelles for WAXS analysis: A solution of micelles of the BCP **8a** was dropped over a freshly cleaved piece of mica (same surface than that previously used for the AFM analysis, so to ensure the formation of the corresponding toroidal and bicontinuous micelles). The drops were placed in different parts of the mica surface to avoid the dropping over the dry film previously formed. The as prepared film-drops were dried 12 h at room temperature (20 °C) and removed with a blade as a white and bright powder. The process was repeated until 75–100 mg of dry sample of micelles were obtained.

#### 4.2.1. Synthesis of the Telechelic P2VP_24_-PPh_2_ (**4**)

In a Young’s tube under dry Ar(g) atmosphere, 0.14 g (0.4 mmol) of **1** were dissolved in 1 mL of toluene with magnetic stirring. The solution was cooled at −78 °C (liquid N_2_/acetone bath) and 4.7 mL (0.8 mmol) of tertBuLi was added slowly (10 min). The resulted cloudy and reddish solution was stirred at −78 °C during 1 h and 30 min at 20 °C. Then, 65 µL (0.4 mmol) of TMEDA was added at 20 °C, and the resulting clear red-brown solution was stirred for 30 min at 20 °C yielding the lithiated phosphine **2**, that was used without any purification (the yield of the lithiation was examined by the reaction of **2** with Cl-SiMe_3_, see details below). Over the stirred solution of **2** at 20 °C, 0.9 mL (8.0 mmol) of 2-vinylpyridine was quickly added. The color of the reaction mixture turned to orange, and the solution was stirred at 20 °C during 2 h. Then, 77 µL (0.6 mmol) of Cl-SiMe_3_ was added, and the reaction mixture was stirred a room temperature until the total disappearance of the orange color (ca. 5 min.). All the volatiles were removed in vacuum and the resulting white solid was dissolved in THF (2–4 mL) and purified by precipitation of the concentrated solutions of THF onto water (×2) and n-hexanes (×2). The resulting white solid was dried for 2 days under reduced pressure at 40 °C.

(**4**): Yield: 0.8 g (95%). ^31^P{^1^H} NMR (CDCl_3_, 25 °C, δ ppm) = −5.5. ^1^H-NMR (CDCl_3_, 25 °C, δ ppm) = 8.6–6.4 (m, Pyridinyl and Ph, 110H); 1.88–1.48 (m, -CH_2_-CH-, 72H); 0.72 (m, -SiCH_3_, 9H). ^13^C{^1^H} NMR (CDCl_3_, 25 °C, δ ppm): 164, 149, 135, 123, 120 (pyridinyl); 41 (br, m, CH_2_-CH-); 0.2 (-SiCH_3_ end group). FT-IR (KBr pellets, cm^−1^): 3024.8 (υ_C-Haromat_.); 2921.3 (υ_C-Hsp3_); 1941.9, 1851.3, 1799.0, 1739.0 (Pyridinil moiety); 1600.9, 1492.8, 1452.0 (υ_C=Caromat._); 755.9, 696.5 (δ_C-H_, pyridinyl moiety). GPC: *M*_n_ = 2980, PDI = 1.15. Elemental analysis (Calc.): C_7.87_ H_7.95_ N Si_0.04_ P_0.04_ (119.1 g/mol) = C: 78.52 (79.4); H: 6.66 (6.70); N: 10.98 (11.75).

The lithiation of **1** with tertBuLi in toluene was checked by the reaction of the lithiated phosphine **2** with Cl-SiMe_3_. Thus, **2** was prepared following the same procedure than that previously described and using the same preparative scale (see above). Then, over a stirred solution of **2** in toluene, 1.2 equiv. of Cl-SiMe_3_ were added observing, after ca. 5 min, the total disappearance of the red-brown color. All the volatiles were removed under vacuum obtaining a clear and colorless viscous oil that was spectroscopically characterized as the phosphine **3**.

(**3**): Yield = 0.135 g (98%). ^31^P{^1^H}-NMR (CDCl_3_, 25 °C, δ ppm) = −5.1. ^1^H-NMR (CDCl_3_, 25 °C, δ ppm) = 7.36–7.35 (m, Ph, 14H); 0.29 (s, -SiCH3, 9H). ^13^C{^1^H} NMR (CDCl_3_, 25 °C, δ ppm): 141.1; 137.8 (d, *J* = 11 Hz); 137.2 (d, *J* = 9 Hz); 133.9 (d, *J* = 19 Hz); 133.5; 132.9 (d, *J* = 18 Hz); 128.8; 128.6; −1.0 Elemental analysis (Calc.): C_21_H_32_PSi (343.52 g/mol); C: 73.42 (73.34); H: 9.29 (9.39).

#### 4.2.2. Synthesis of Block Copolymers PTFEP-b-P2VP (**8a**–**c**)

The presented experimental procedure (see below) corresponds to BCP **8a**. Block copolymers **8b** and **8c** were prepared in a similar manner, adjusting the amount of Cl_3_P=N-SiMe_3_ (**6**) and NaOCH_2_CF_3_ to the desired block ratio.

(In a Young’s tube under Ar(g) atmosphere). To a stirred solution of **4** (*M*_n_ = 2980; 0.30 g, 0.10 mmol of –PPh_2_) in 5 mL of degassed CH_2_Cl_2_, 28 mg of C_2_Cl_6_ (0.12 mmol) was added. The solution was stirred at 20 °C during 72 h. The ^31^P{^1^H}-RMN revealed the disappearance of the signal at −5.5 ppm (**4**) and the appearance of a new signal at 65.3 ppm corresponding to the chlorinated polymer P2VP-PPh_2_Cl_2_ (**5**) that was used in situ without further purifications. Then, Cl_3_P=N-SiMe_3_ (0.44 g, 2.0 mmol) was added, and the mixture was stirred at 20 °C during 18 h. The ^31^P{^1^H}-RMN revealed the disappearance of the signals at −54.31 ppm (Cl_3_P=N-SiMe_3_) and 65.3 ppm (-PPh_2_Cl_2_), and the appearance of a new signal at −18.3 ppm corresponding to the [N=PCl_2_]_n_ block of the block copolymer **7** ([N=PCl_2_]-*b*-P2VP). All the volatiles were removed under vacuum and the resulting block copolymer **7** was dissolved in 5 mL the THF and treated with an excess (20%) of NaOCH_2_CF_3_ in THF (NaOCH_2_CF_3_ was prepared by reaction of HOCH_2_CF_3_ with NaH at room temperature in THF). The ^31^P{^1^H}-RMN after 18 h of reaction at 20 °C, revealed the disappearance of the signal at −18.3 ppm ([N=PCl_2_]) and the appearance of a new signal at −6.8 ppm corresponding to the PTFEP block ([N=P(O_2_CH_2_CF_3_)_2_]_n_). Then, all the volatiles were removed in vacuum and the resulting white solid (**8a**) was dissolved in THF (2-4 mL) and purified by precipitation of the concentrated solutions of THF onto water (x2), and *n*-hexanes (x2). The resulting white solid was dried for 2 days under reduced pressure at 40 °C.

(**8a**): Yield: 0.42 g (54%). ^31^P{^1^H}-NMR (CDCl_3_, 25 °C, δ ppm) = −6.7 (N=P(OCH_2_CF_3_)_2_). ^1^H NMR (CDCl_3_, 25 °C, δ ppm) = 8.9–6.2 (m, Pyridinyl and Ph, 110H); 4.6 (m, -OC*H*_2_CF_3_, 88H); 1.98–1.26 (m, -CH_2_-CH-, 72H); 0.62 (m, -SiCH_3_, 9H). ^13^C{^1^H}-NMR (CDCl_3_, 25 °C, δ ppm): 163, 144, 132, 125, 120 (pyridinyl aromatic pendants); 128.4 (m, N=P(OCH_2_*C*F_3_)_2_); 63.1 (m, *J* = 83.2 Hz, N=P(O*C*H_2_CF_3_)_2_); 40 (br, m, CH_2_-CH-); 0.2 (-SiCH_3_ end group). ^19^F NMR (CDCl_3_, 25 °C, δ ppm) = −77.4 (s, br). FT-IR (KBr pellets, cm^−1^): 3022.3 (υ_C-Haromat._); 2922.6 (υ_C-Hsp3_); 1944.9, 1853.1, 1796.3, 1738.7 (aromatic); 1604.9, 1494.6, 1454.3 (υ_C=Caromat._); 1287.3 (υ_PO-C_); 1170.4 (υ_PN_); 1082.0 (υ_P-OC_); 756.9, 694.3 (δ_C-H_, pyridinyl). GPC: *M*_n_ = 20500, PDI = 1.15.

(**8b**): Yield: 0.91 g (52%). ^31^P{^1^H}-NMR (CDCl_3_, 25 °C, δ ppm) = −6.6 (N=P(OCH_2_CF_3_)_2_). ^1^H NMR (CDCl_3_, 25 °C, δ ppm) = 8.9–6.2 (m, Pyridinyl and Ph, 110H); 4.8 (m, -OC*H*_2_CF_3_, 256H); 1.98–1.26 (m, -CH_2_-CH-, 72H); 0.64 (m, -SiCH_3_, 9H). ^13^C{^1^H} NMR (CDCl_3_, 25 °C, δ ppm): 162, 147, 135, 122, 119 (pyridinyl aromatic pendants); 128 (m, N=P(OCH_2_*C*F_3_)_2_); 62 (m, *J* = 83.2 Hz, N=P(O*C*H_2_CF_3_)_2_); 41 (br, m, CH_2_-CH-); 0.2 (-SiCH_3_ end group). ^19^F-NMR (CDCl_3_, 25 °C, δ ppm) = −78.2 (s, br). FT-IR (KBr pellets, cm^−1^): 3022.3 (υ_C-Haromat._); 2922.6 (υ_C-Hsp3_); 1944.9, 1853.1, 1796.3, 1738.7 (aromatic); 1605.3, 1488.6, 1443.2 (υ_C=Caromat._); 1289.3 (υ_PO-C_); 1174.1 (υ_PN_); 1080.1 (υ_P-OC_); 759.9, 684.1 (δ_C-H_, pyridinyl). GPC: *M*_n_ = 42600, PDI = 1.18.

(**8c**): Yield: 1.23 g (452%). ^31^P{^1^H} NMR (CDCl_3_, 25 °C, δ ppm) = −6.9 (N=P(OCH_2_CF_3_)_2_). ^1^H-NMR (CDCl_3_, 25 °C, δ ppm) = 8.6–6.0 (m, Pyridinyl and Ph, 110H); 4.7 (m, -OC*H*_2_CF_3_, 392H); 2.0–1.20 (m, -CH_2_-CH-, 72H); 0.54 (m, -SiCH_3_, 9H). ^13^C{^1^H}-NMR (CDCl_3_, 25 °C, δ ppm): 146, 134, 122, 120 (pyridinyl aromatic pendants); 128 (m, N=P(OCH_2_*C*F_3_)_2_); 62 (m, *J* = 83.2 Hz, N=P(O*C*H_2_CF_3_)_2_); 41 (br, m, CH_2_-CH-). ^19^F NMR (CDCl_3_, 25 °C, δ ppm) = −76.5 (s, br). FT-IR (KBr pellets, cm^−1^): 3022.3 (υ_C-Haromat._); 2922.6 (υ_C-Hsp3_); 1607.1, 1486.4 (υ_C=Caromat._); 1286.2 (υ_PO-C_); 1175.3 (υ_PN_); 1084.2 (υ_P-OC_). GPC: *M*_n_ = 61200, PDI = 1.18.

## Figures and Tables

**Table 1:** Molecular weight data and yields of PTFEP-*b*-P2VP block copolymers **8a**–**c**.

BCP	*M*_n_ (g/mol) ^1^	PDI ^1^	*M*_n_ (g/mol) ^2^	DP[2](n/m)	φ_PTFEP_^3^	Yield (%)
**8a**	20,500	1.15	7870	24/22	0.58	54
**8b**	42,600	1.18	18,075	24/64	0.80	52
**8c**	61,200	1.18	26,340	24/98	0.86	45

^1^ Determined by GPC (conventional calibration vs. polystyrene standards). ^2^ Determined by comparing the ^1^H NMR integration of the resonances associated with PTFEP and P2VP blocks. ^3^ Volume fraction of PTFEP block calculated using the following density values (g/cm^3^) for both blocks: 1.665 (PTFEP) [51] and 1.09 (P2VP) [52].

## Supplementary Materials

The following are available online: Characterization details of BCPs **8a**–**c**. DLS data related to the self-assembly studies.
