# Supplementary material for: Cylindrical Micelles by the Self-Assembly of Crystalline-b-Coil Polyphosphazene-b-P2VP Block Copolymers. Stabilization of Gold Nanoparticles
Source: Molecules. 2019 May 7;24(9):1772. doi: 10.3390/molecules24091772 (PMC6539542; doi:10.3390/molecules24091772)
Supplement: Supplementary file 1 [file molecules-24-01772-s001.pdf]

**Supporting Informatio for**

# Cylindrical Micelles by the Self-Assembly of Crystalline-b-Coil Polyphosphazene-b-P2VP Block Copolymers. Stabilization of Gold Nanoparticles

**Maria de los Angeles Cortes [1], Raquel de la Campa [2], Maria Luisa Valenzuela [3], Carlos Díaz [1], Gabino A. Carriedo [2] and Alejandro Presa Soto [2,*]**

[1] Department of Chemistry, School of Chemistry, University of Chile, La Palmeras, 3425, Nuñoa, Casilla 653, 7800003 Santiago de Chile, Chile; mariadelosangeles.cortesc@gmail.com (M.d.l.A.C.); cdiaz@uchile.cl (C.D.)

[2] Department of Organic and Inorganic Chemistry (IUQOEM), School of Chemistry, University of Oviedo, Julián Clavería s/n, 33006 Oviedo, Spain; delacampafernandez@yahoo.es (R.d.l.C.); gac@uniovi.es (G.A.C.)

[3] University Autónoma de Chile, School of Engineering, Institute of Applied Chemistry Science., Inorganic Chemistry and Molecular Material Center, Av. El Llano Subercaseaux, 2801, 8900000 Santiago de Chile, Chile; maria.valenzuela@uautonoma.cl (M.L.V)

* Correspondence: presaalejandro@uniovi.es (A.P.S.); Tel: +34-985-10-2986

1. NMR spectra of P2VP-PPh2 (**4**)

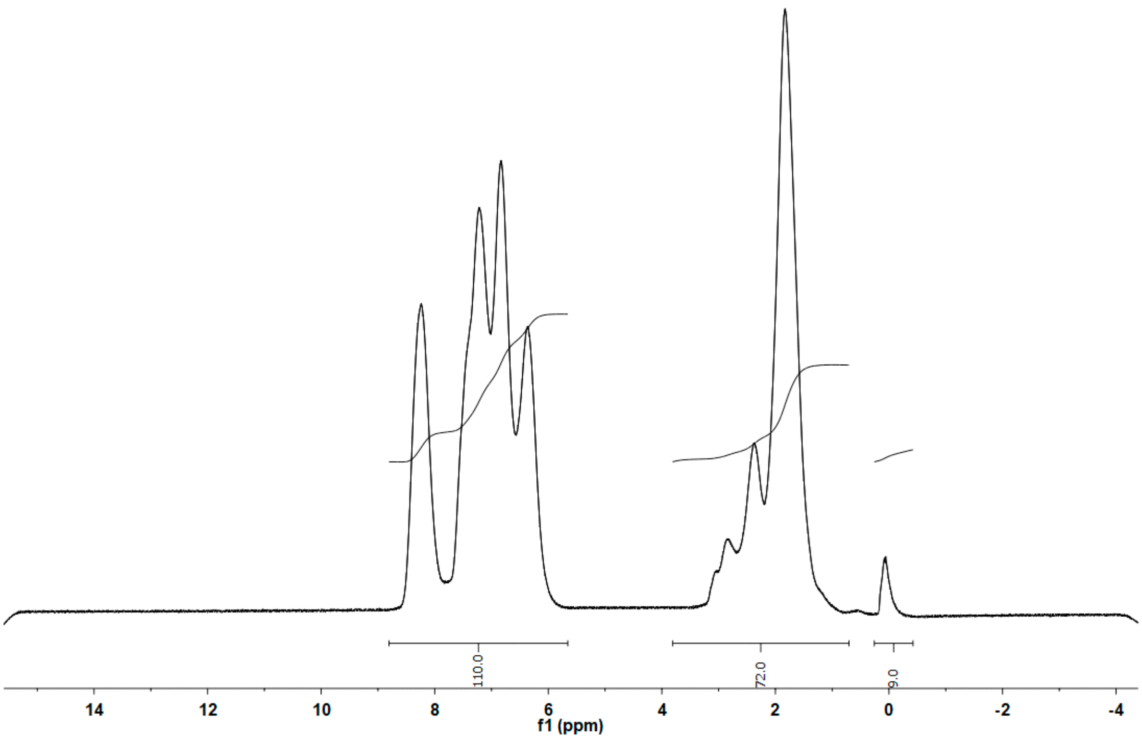


**Figure S1**. $^{1}$H-NMR ($CDCl_3$)

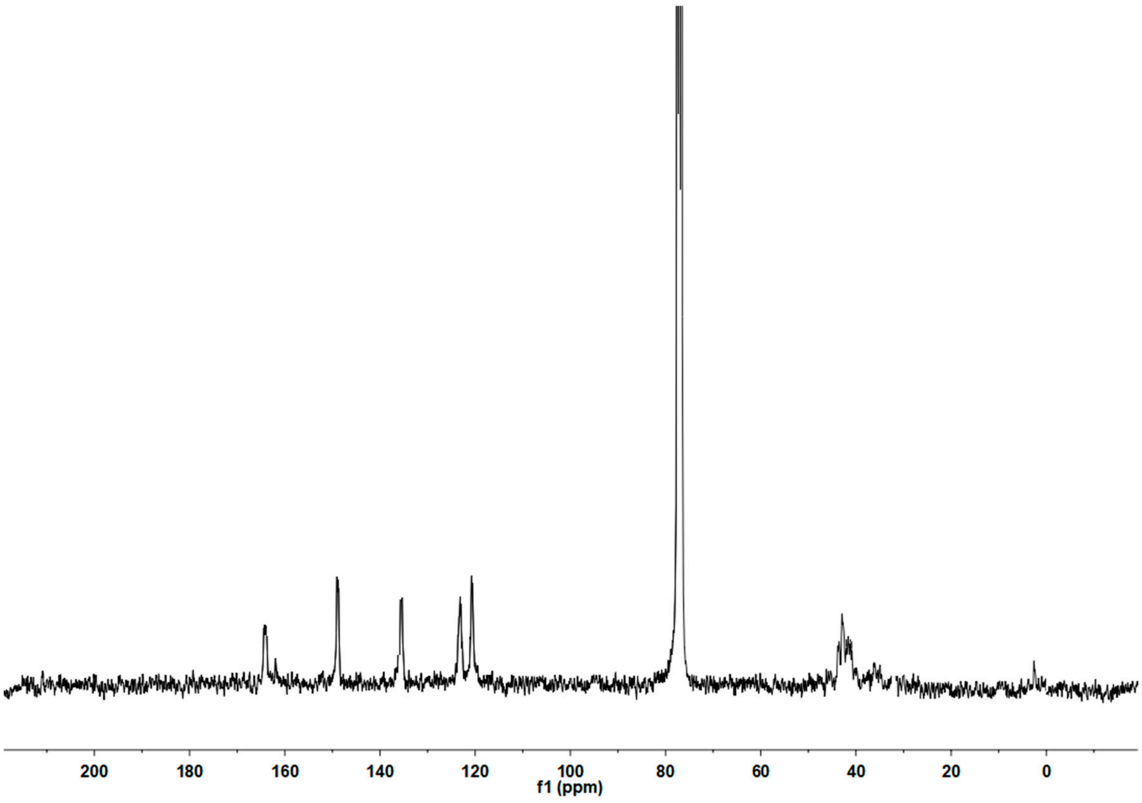


**Figure S2**. $^{13}$C-{$^{1}$H}-NMR ($CDCl_3$)

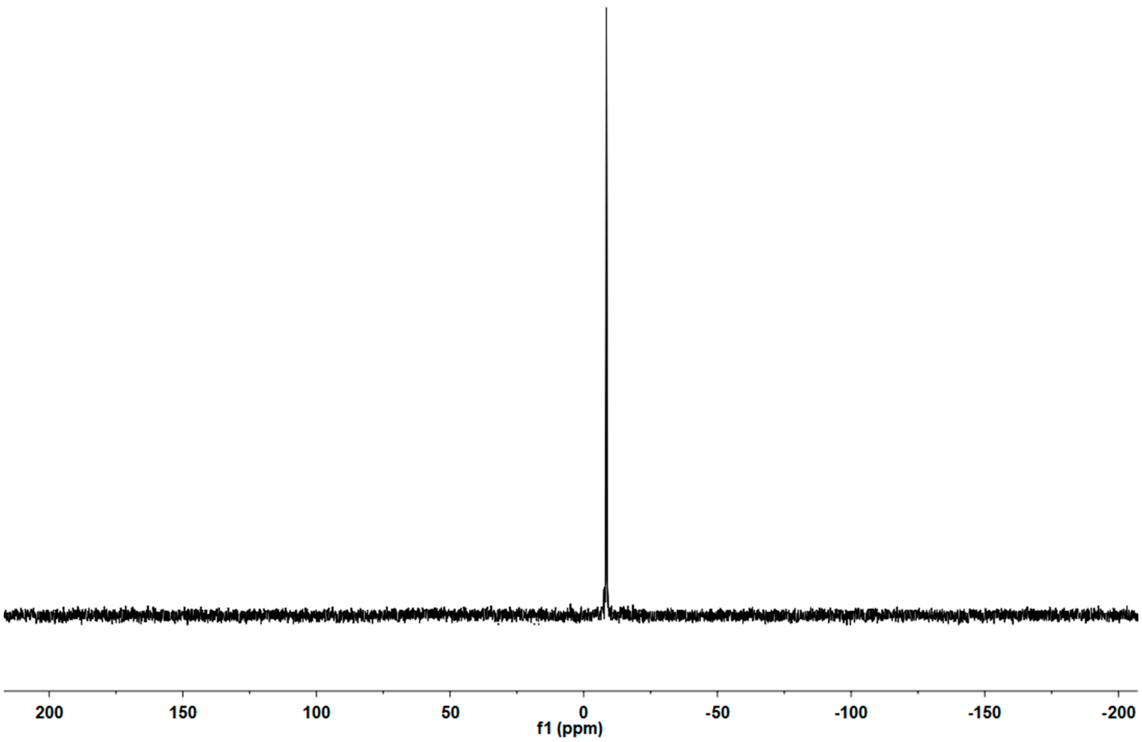


**Figure S3**. $^{31}P$-{$^{1}H$}-NMR ($CDCl_3$)

2. NMR spectra of P2VP-$PPh_2Cl_2$ (**5**)

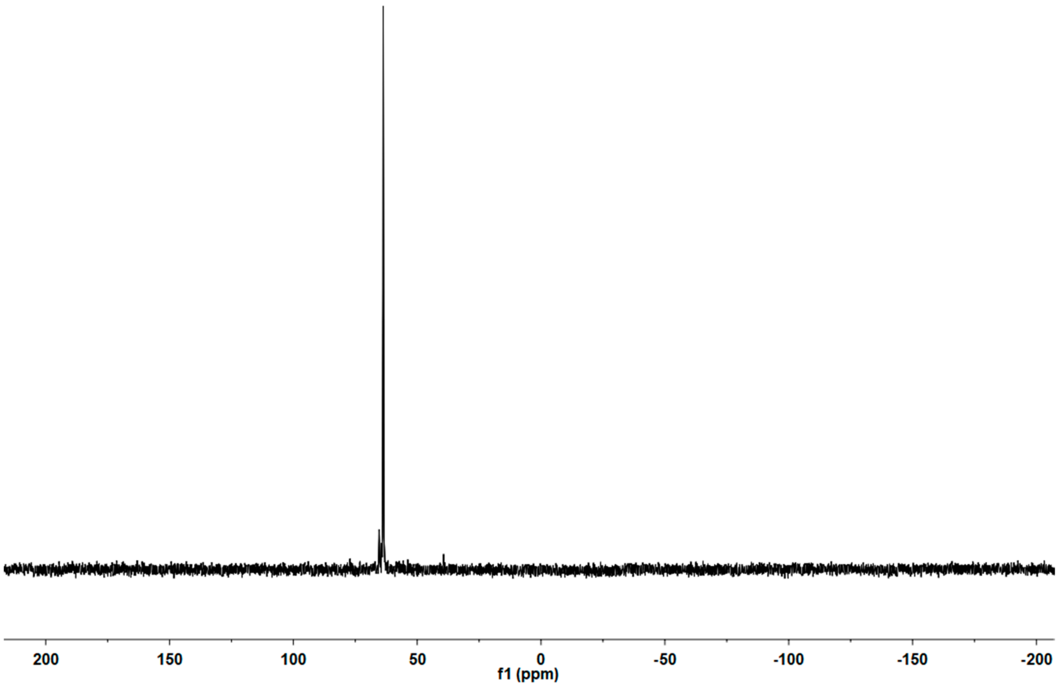


**Figure S4**. $^{31}P$-{$^{1}H$}-NMR ($CH_2Cl_2$ with external reference of $D_2O$)

3. NMR spectra of PTFEP-*b*-P2VP (**8a**)

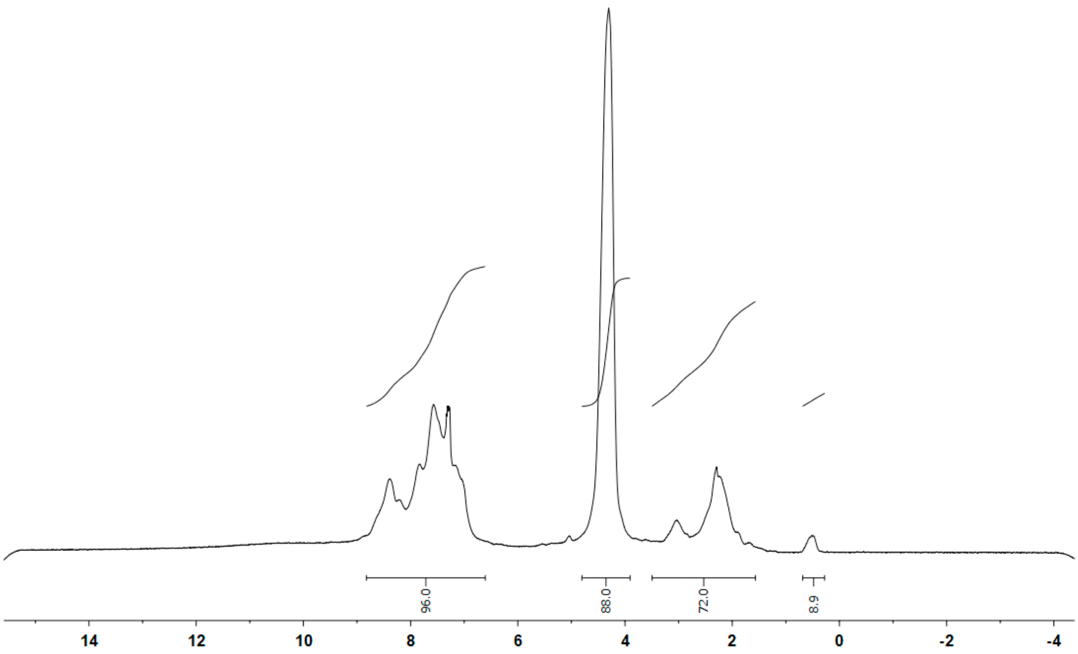


**Figure S5**. $^{1}$H-NMR ($CDCl_3$)

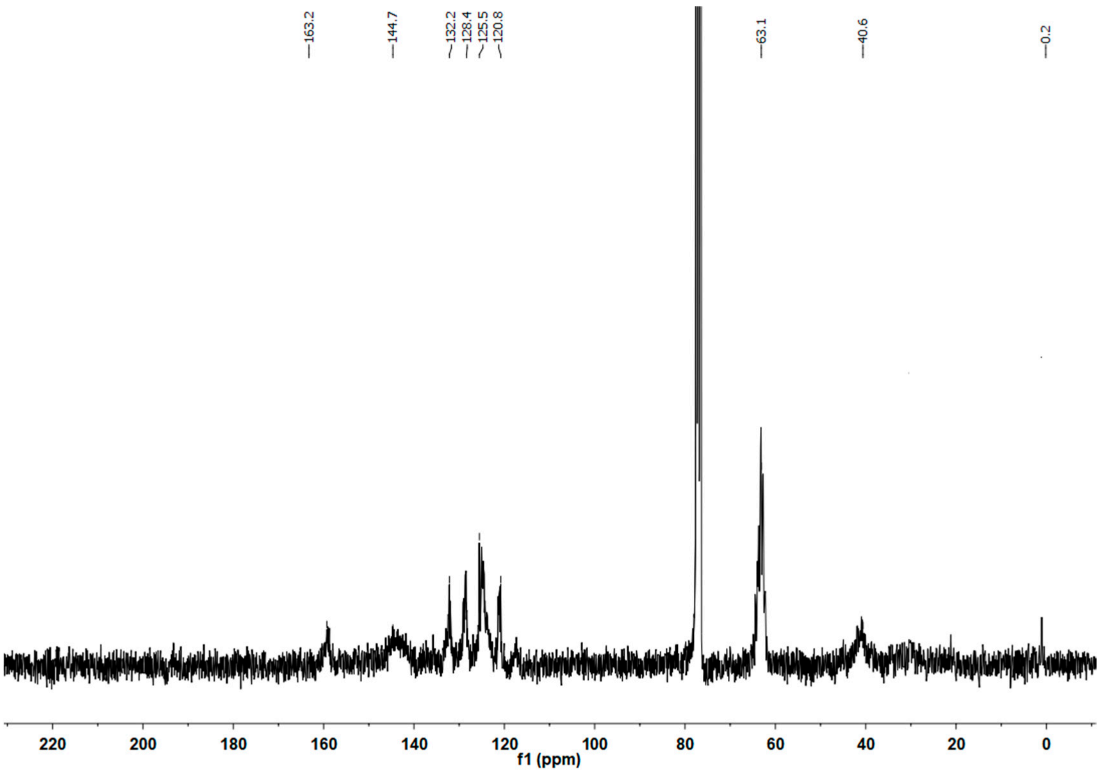


**Figure S6**. $^{13}$C-{$^{1}$H}-NMR ($CDCl_3$)

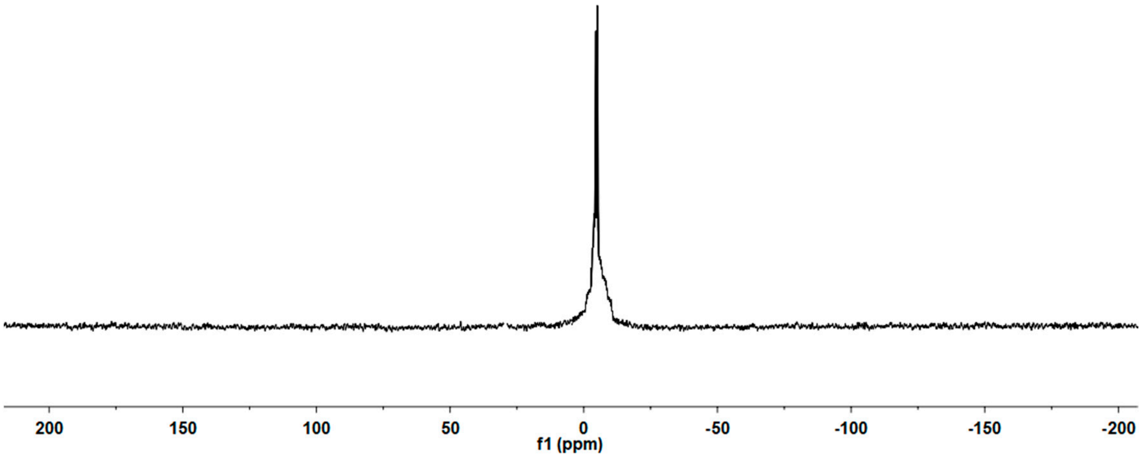


**Figure S7**. $^{31}P$-{$^{1}H$}-NMR ($CDCl_3$)

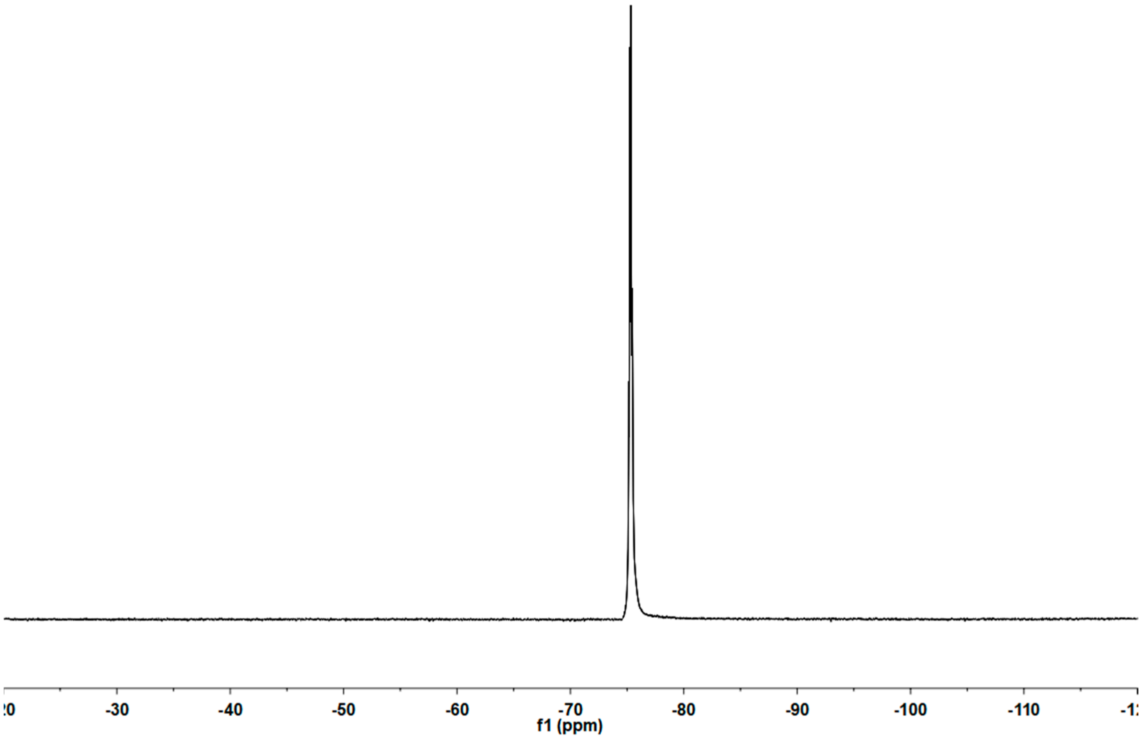


**Figure S8**. $^{19}F$-{$^{1}H$}-NMR ($CDCl_3$)

4. DLS data of the self-assembly studies of PTFEP-*b*-P2VP (**8a-c**)

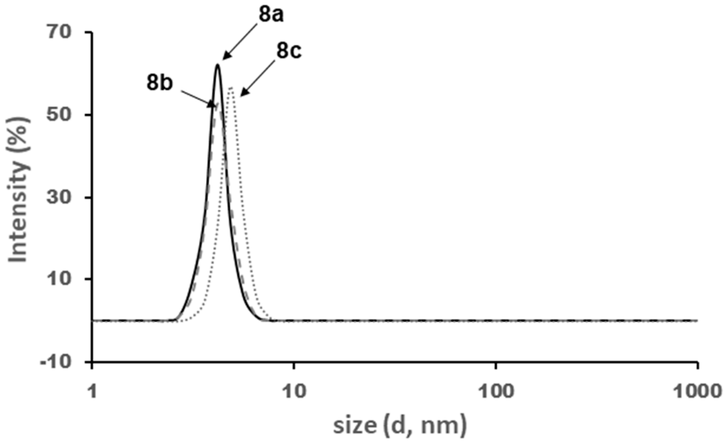


**Figure S9**. DLS of BCPs **8a-c** in THF at 0.5 mg/mL

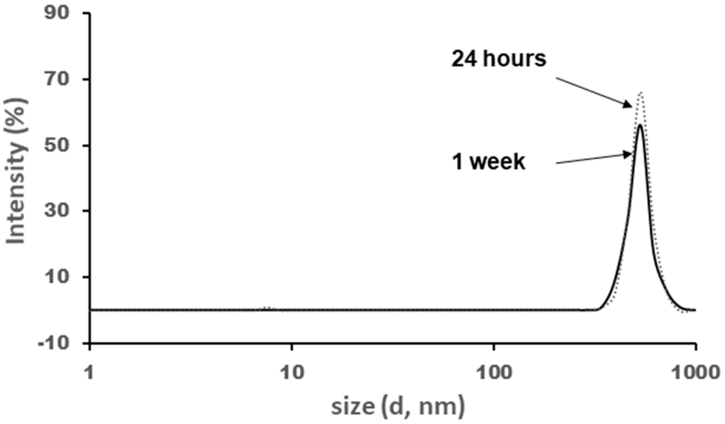


**Figure S10**. DLS of a solution of BCP **8a** at different times (24 h and 1 week) after the addition of 50 % vol. of 2-propanol over a 0.5 mg/mL THF solution of **8a** (addition rate = 1 drop / 5 sec).

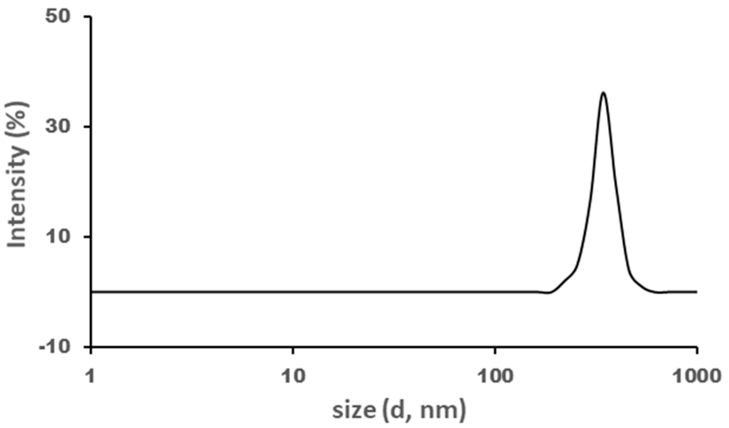


**Figure S11**. DLS of a solution of BCP **8a** after the addition of 100 % vol. of 2-propanol over a 0.5 mg/mL THF solution of **8a** (addition rate = 1 drop / 5 sec).

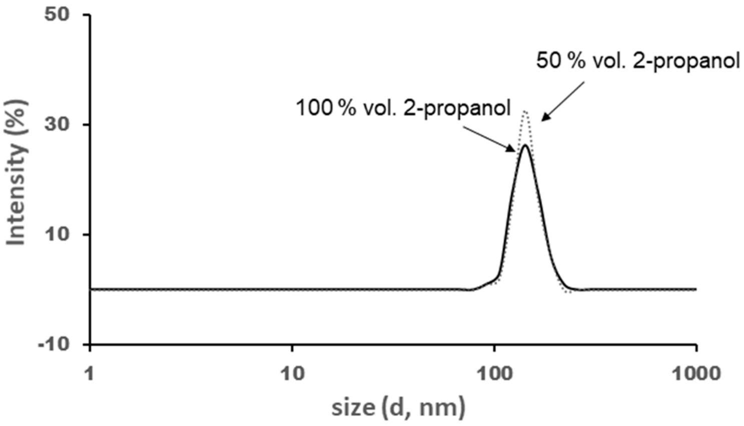


**Figure S12**. DLS of solutions of BCP **8a** after the addition of 50 and 100 % vol. of 2-propanol over a 0.5 mg/mL THF solution of **8a** (addition rate = 1 step).
